# Keys to successful implementation of a French national quality indicator in health care organizations: a qualitative study

**DOI:** 10.1186/s12913-016-1794-7

**Published:** 2016-10-06

**Authors:** Mathias Waelli, Marie-Léandre Gomez, Claude Sicotte, Adrian Zicari, Jean-Yves Bonnefond, Philippe Lorino, Etienne Minvielle

**Affiliations:** 1 EA 7438 MOS, EHESP (French School of Public Health), Rennes, France; 2 ESSEC Business School, Cergy Pontoise, France; 3 Montreal University, Montreal, Canada; 4 CNAM (International Institute of Management), Paris, France; 5 Gustave Roussy Institute, Villejuif, France; 6 EHESP, 8 rue Maria Helena Vieira Da Silva – 75014, Paris, France

**Keywords:** Implementation, Quality indicators, Health care organizations, Anesthesia

## Abstract

**Background:**

Several countries have launched public reporting systems based on quality indicators (QIs) to increase transparency and improve quality in health care organizations (HCOs). However, a prerequisite to quality improvement is successful local QI implementation. The aim of this study was to explore the pathway through which a mandatory QI of the French national public reporting system, namely the quality of the anesthesia file (QAF), was put into practice.

**Method:**

Seven ethnographic case studies in French HCOs combining in situ observations and 37 semi-structured interviews.

**Results:**

A significant proportion of potential QAF users, such as anesthetists or other health professionals were often unaware of quality data. They were, however, involved in improvement actions to meet the QAF criteria. In fact, three intertwined factors influenced QAF appropriation by anesthesia teams and impacted practice. The first factor was the action of clinical managers (chief anesthetists and head of department) who helped translate public policy into local practice largely by providing legitimacy by highlighting the scientific evidence underlying QAF, achieving consensus among team members, and pointing out the value of QAF as a means of work recognition. The two other factors related to the socio-material context, namely the coherence of information systems and the quality of interpersonal ties within the department.

**Conclusions:**

Public policy tends to focus on the metrological validity of QIs and on ranking methods and overlooks QI implementation. However, effective QI implementation depends on local managerial activity that is often invisible, in interaction with socio-material factors. When developing national quality improvement programs, health authorities might do well to specifically target these clinical managers who act as invaluable mediators. Their key role should be acknowledged and they ought to be provided with adequate resources.

## Background

Recently, health authorities in many countries have launched public reporting campaigns to improve the quality of care based on validated quality indicators (QIs) QIs should be understood here as statistical measures that assess output or processes. The premise is that public disclosure of QI scores will increase transparency and encourage quality improvements in health care organizations (HCOs) [1–3].

Quality improvements necessitate the development not only of valid QI measures but also the means to enable the introduction of corrective interventions that befit the local context [4]. Indeed, QI measurement alone does not ensure corrective interventions that will improve QI scores [5]. Accounting is a matter of “style” rather than “substance” [6]. Thus, the value of a QI depends on its adoption by HCO staff and in the changes in practice or work organization that will improve quality. For instance barriers arising during implementation may lead to suboptimal QI assessment (lower scores than expected) [6] and become divorced from operational activities or induce unwelcome effects (for example, defensive responses, a decrease in quality, and gaming practices [7–9]).

A recent review on causal pathways between performance measure use and effect stressed the need to develop research in the area of QI implementation [10]. In a national public reporting system a mandatory assessment tool (the QI) is used to acquire quality improvement results, either at the macro level to support investment decisions or at the micro level to help frontline workers improve service [11]. This contrasts with the usual global way local QI implementation is considered in terms of total quality management tools or continuous quality improvement approaches and strategies [12–15].

According to the emerging field of the implementation sciences [16], this study aims to explore the pathway through which measurement tools come to be operationalized in practice.

To do so we link empirical findings to a pragmatic approach reminiscent of cultural-historical activity theory [17]. This approach focuses on the impact of a tool (here, a QI) on human actions and practice. A QI does not operate in an isolated context but within an action scheme (i.e. within an interpretative framework described by an implicit “how to use” guide for the tool) that is constantly being updated through individual practitioners’ experiences.

To understand the factors influencing effective QI use, and the interaction between these factors and the local context, we undertook a qualitative analysis of a QI - the quality of the anesthesia file (QAF).- over a two-year period (2011–2013).

## Method

### Choice of QI

In order to focus on the issue of implementation in a local context (rather than its relevance), we selected a QI that had characteristics that were in strong alignment with professional practice. Anesthestists have developed a strong culture of data traceability [18] and consider the anesthesia file as representing a major quality issue. Their work requires planning [19] and collaboration [20, 21]. This is especially true in France where any patient planning to have surgery must consult an anesthetist one month prior to the surgery date, as well as a few hours before surgery at admission. These consultations are often made by different doctors working in a large team. Thus, anesthetists’ activity relies heavily on the data included in a patient’s anesthesia file. The quality of the anesthesia file (QAF) is one of the 62 QIs that are part of the French national public reporting system introduced in 2006 and coordinated by the French Ministry of Health and French National Authority for Health (*Haute Autorité de Santé,* HAS) [22]. The anesthesia file is part of the patient file, is used primarily by anesthesia physicians and nurse anesthetists (sometimes by surgeons), and remains a paper document in most French HCOs.

The QAF assesses whether certain elements of information using 13 key criteria (Table 1) are present in the anesthesia file over 3 distinct periods (pre-operative, per-operative, and post-operative). The 13 criteria were selected as a result of research studies and validated by professionals, members of the French Anesthesia and the Intensive Care Society (*Société Française d’Anesthésie et de Réanimation,* SFAR), [23]. The premise is that information shared among anesthetists provides higher quality care.

**Table 1:** Key elements of QAF

	Quality indicator description form
Definition	This quality indicator, expressed as a score, evaluates the state of the anesthesia file. For every file chosen at random, a quality score between 0 and 1 is calculated based on a maximum of 13 criteria. The closer the score is to 1, the higher the estimated quality of the file.
Criteria	1. Patient identification on every page of the file Pre-anesthesia 2. Anesthetist named in file (pre-anesthesia consultation or visit) 3. Records from pre-anesthesia visit attached (pre-anesthesia visit) 4. Ongoing medical treatment (or lack of treatment) recorded in file (pre-anesthesia consultation or visit) 5. Evaluation of anesthesia risk recorded in file (pre-anesthesia consultation or visit) 6. Type of anesthesia proposed to patient recorded in file (pre-anesthesia consultation or visit) 7. Pre-anesthesia evaluation of access to upper airways recorded in file (pre-anesthesia consultation or visit) Anesthesia during intervention 8. Anesthetist present during intervention named in file 9. Technical approach for access to upper airways recorded in file (if applicable) Immediate post-anesthesia care 10. Anesthetist present at post-intervention named in file (if applicable) 11. Anesthetist’s signature authorizing patient’s discharge from recovery room in file (if applicable) 12. Post-intervention medical prescriptions recorded (if applicable) Throughout 13. Space for recording peri-anesthesia incidents or accidents
Sample Size	A sample size of 60 files in each health care organization is used to calculate an average score
Type of Indicator	Process indicator.
	Composite indicator.
	Risk adjustment: no.

Since 2008, each of the 1300 acute care HCOs in France have had to evaluate their compliance with the 13 criteria in a random sample of 60 files for patients having undergone surgery the preceding year. A composite score is calculated and expressed as a percentage of average score of the 60 files. The national target as defined in 2009 is 80 %. Assessment was conducted yearly until 2011 and was then reduced to every 2 years to reduce the burden of data collection.

### Case studies

We attended three 3-h work sessions organized by the HAS and SFAR to learn about QAF design. We retrieved the minutes of earlier meetings as well as official documents and reports to acquire the background knowledge needed for our case studies (2012–2013). In order to learn more about the local contexts that could affect how the QAF was implemented we selected 6 diverse HCOs (Table 1), distinct in size, types (teaching/nonteaching hospitals), status (public/private), and location. Each HCO works as a “polar case” [24], providing an opportunity to explore significant phenomena. Before contacting the individual anesthesia teams we first sent a formal e-mail to the management of each of the HCOs outlining our research objectives and to obtain institutional approval.

In order to focus on the implementation process without normative consideration, in a first step our case selection did not take into account the two available QAF scores (2010/2011) obtained by the HCOs. Two of the six HCOs (A & E) had in fact achieved excellent QAF scores in their last assessment. The four other HCOs had slightly lower scores but nevertheless showed marked progress. In a second step, a contrasting HCO case study was selected whose QAF score had fallen by 24 % between 2010 and 2011 (G in Tables 1 and 2).

**Table 2:** Case studies in 2012

HCO description			Study methods		
Type (Location)		Beds Anesthetists Nurses Cases/year (n)	Observation	Informal discussion	In-depth Interview
A	Private, not-for- profit (Paris region)	339	Yes	Yes	1 Chief anesthetist (2×)
		14			1 Anesthetist
		20			1 Nurse anesthetist
		8470			1 Physician in charge of QIs
B	Public, university hospital (Paris)	753	Yes	Yes	1 Chief anesthetist (2×)
		36			3 Anesthetists
		46			3 Nurse anesthetists
		17690			1 Quality manager
C	Public, university hospital (West of France)	1318	No	No	1 Medical coordinator
		50			1 Chief anesthetist
		90			1 Anesthetist
		31460			1 Nurse anesthetist
D	Public (South of France)	617	Yes	Yes	1 Medical coordinator
		15			1 Chief anesthetist
		25			1 Anesthetist
		12730			2 Nurse anesthetists
					1 Quality manager
					1 Surgeon
E	Private for Profit (East of France)	162	Yes	Yes	1 Chief anesthetist
		8			1 Nurse anesthetist
		16			1 Head nurse
		15300			1 Quality manager
F	Private for Profit (Paris)	237	No	No	1 Chief executive officer
		8			1 Chief anesthetist
		NA			1 Physician in charge of information systems
		13400			1 Nurse anesthetist
G^a^	Public (East of France)	355	Yes	Yes	1 Chief anesthetist
		6			2 Anesthetists
		10			1 Nurse anesthetist
		6522			

*NA* not available

^a^Contrasting case

### Data collection

In each HCO observations were made and semi-structured interviews were held by at least 2 researchers in order to ensure standard reporting. Observations (1–5 days) involved tracking the anesthesia file. The anesthesia file was approached as “work practice centered” as noticed elsewhere [25]. It allowed us to identify the different professional actors interacting with the anesthesia file. We observed how it was completed and used in the anesthesia consultation offices and in the operating room area in five of the seven HCOs. During our observations staff were asked what they knew about the QAF and what they thought about any changes that had been introduced since the QAF has been implemented. Several informal “hallway” interviews were also carried out during this time. We conducted 37 semi-structured interviews with staff involved in the QAF implementation and/or data collection (4 to 9 members/HCO according to HCO type) (Table 1): 18 anesthetists (all full-time), 10 nurses, 3 quality managers, and 6 stakeholders (e.g., head of Information Systems Department). The interview guide developed by the researchers was used to question their knowledge of QAF and the local aspects of QAF implementation. Overall, 35/37 interviews were digitally recorded and fully transcribed. Two interviews were not audio recorded, but we took detailed notes during interviews. Documents (whether in house or not) were collected on the QAF scores and improvement initiatives in each HCO.

### Data analysis

The data from the case studies were analyzed in several ways. Following an inductive approach, we first used open coding to summarize segments of data. In a second step, as data collection progressed, the codes with common elements were merged into categories in line with the issue of QAF implementation (use of electronic health record, improvement actions, interpersonal ties…). These categories were then compared through cross case analysis during monthly meeting with the research team, allowing us to identify factors influencing QAF implementation according to the local context. At each step of the data analysis validity was increased by the use of multiple data sources [26].

## Results

### Limited perceived use of QAF and quality Improvements

Very few anesthetists and nurses were aware of the QAF. In general, QAF scores were not perceived to hold any special relevance to how well an anesthesia unit functioned: “*I flicked through the information on the indicator and went on to my next task*” (Chief anesthetist, HCO A); “*The anesthesia teams do not show much interest in the yearly scores*” (Quality manager, HCO B). Our observations in the operating area underscored these comments. On presenting our study to the anesthesia teams, we nearly always had to explain the meaning of QAF. Awareness of QAF was restricted to staff members who had participated in data collection (the chief anesthetist in HCO D had even forgotten despite his involvement) and, in the contrasting case (HCO G), to the chief anesthetist who was the only person to mention QAF scores during our visit.

However staff members were largely involved in quality improvement initiatives to meet QAF criteria. Anesthetists and nurse anesthetists in HCO D considered the changes made to the anesthesia file to be improvements in practice but never mentioned that they considered the QAF criteria to be at the root of these changes. This was indeed confirmed by the quality manager. Over the course of our study the QAF scores across the first 6 HCOs improved steadily. Changes were made to the anesthesia file in all 7 HCOs to facilitate documenting criteria, ensure better quality of information, and achieve higher QAF scores. In HCO G, the newly appointed chief anesthetist planned to use the QAF to underscore work organization problems. In HCO B, a task force was tackling difficult intubation, an important but often poorly documented safety criterion..In HCO C, two boxes restricting choice (standard risk or high risk) were added to the blank space in the last section of the file.

### Key role of clinical managers in QAF implementation

The anesthetists in charge of data collection in all 7 HCOs developed an interest in QAF. In HCO C, the anesthetist who collected data on all 60 files decided to analyze the data himself, made a note of potential problems, and submitted his analysis for discussion with other staff. In HCO D, whilst collecting data, the chief anesthetist noted that the QAF scores reflected differences in post-anesthesia care in facilities at two different sites (Table 1, criteria 10–12) (criterion 10 was documented in 40 % of files at the smaller site versus 98 % at the larger site). The physician’s signature authorizing patient discharge from the recovery room was present in only 60 % of files at the smaller site. “*I was not aware that documenting was poor at this site* (…). *The process enabled us to evaluate our work better. If there had been a court case and a judge had discovered from the file that the patient had been discharged without the physician’s signature, where would we have been?” (Chief anesthetist, HCO D)*Participating in data collection is a means not only of producing statistics for external accountability but also of generating awareness of the quality of the work done. An anesthetist in HCO D, who was proud to discover that the unit’s files were well kept, was motivated to devote her time and energy to the implementation of improvement initiatives to meet QAF criteria.

All the chief anesthetists and some of the anesthetists involved in data collection were also involved in promoting corrective actions. The reasoning adopted by these clinical managers (chief anesthetists) and the improvement initiatives they proposed to meet QAF objectives were seen to be legitimate as they were in line with local concerns and needs. We identified 3 lines of reasoning:

(i) Scientific evidence: Health professionals are more likely to accept quality improvement initiatives if they are supported by scientific evidence. The QAF score in HCO A was poor because the criterion for patient discharge from the recovery room (criterion 11) was not completed because it was inconvenient (distance from operating area). Despite the poor score, the anesthetists remained adamantly against signing the discharge sheet. It was only when the chief anesthetist pointed out a published randomized trial indicating that the signature had a positive impact on quality that the staff agreed to support the improvement initiative.
(ii) Consensus: Scientific evidence needs to be supported by consensus. *“Even if the corrective action seems obvious or is evidence based, collective decision-making is needed in order to avoid resistance*” (Chief anesthetist, HCO A).
(iii) Work recognition: Because QAF scores are made public they are a means for the hospital and staff to gain recognition. Recognition was particularly important to practitioners working in private sector hospitals competing for clients: “*If you know yourself that you are doing a good job, you want others to know as well. You’re not going to shoot yourself in the foot*” (Chief anesthetist, HCO E).

### The major role of information system

A major issue for QAF implementation appeared to be the use of an electronic information system. Interviewees in all 7 HCOs considered to be a positive relationship between the spread of electronic information systems and the completeness of the anesthesia files. The use of software had many advantages. For instance, it could bring file completion to a halt after an inappropriate answer; it could complete information (e.g. replace physicians’ signed initials by their full signature (HCO A) or declare peri-anesthetic incidents by default (HCO B). The IT system in HCO C included the initial consultation and the final document in the patient record to improve QAF score. However, although digitalizing may facilitate data collection, there is little incentive to make any changes if the existing system is coherent and paper sheets are easy to trace. In 4 HCOs, the anesthesia file was still a paper file. The HCO G was encountering technical difficulties but was planning on developing an electronic information system with compulsory steps (Table 3).

**Table 3:** Summary of the qualitative analysis in the 7 HCOs

HCO	Local manager	Digitalization of anesthesia record	Professional ties
A	Chief anesthetist	Yes	Very young team
B	Chief anesthetist	No	Diffusion of best practices difficult because of large anesthesia team
C	Chief anesthetist + 2 anesthetists	No	Diffusion of best practices difficult because of large anesthesia team
D	Chief anesthetist + 1 anesthetist	No	New and merged facilities meant that many experienced anesthetists left whilst the improvement assessments were in progress, leaving work to a less experienced team
E	Chief anesthetist	Yes (information system designed and installed by chief anesthetist. Both adapted and adaptable to user needs)	Private sector anesthetists caring little for institutional improvements apart from the chief anesthetist
F	Chief anesthetist	No	Team little concerned with institutional improvements apart from the chief anesthetist who identified with patients and showed high commitment to the steps taken to improve quality
G	Chief anesthetist	Yes (technical difficulties; junior anesthetists had to enter senior anesthetists’ written notes on their tablets but, as wi-fi did not work in the hospital wings, they had to reconvene in the operating rooms).	Strained relationship between the senior anesthetists resisting introduction of new technologies and practices and the chief anesthetist seeking compliance with QAF criteria

### Imbrication of social and material constraints during QAF implementation

The improvement of QAF scores is not a mechanical result of a new technology’s implementation. In HCO E, the chief anesthesiologist had to design a flexible software that met both the objectives of QAF and the specific needs and values of the anesthesia team. “*Physicians will agree to changes in their routines as long as they do not lose time, I update the software constantly*”. Adjustments are made mutually between human actions and technologies. Changes to local practice always require stakeholder involvement: “expert systems can produce such actions only to the extent that the people intended to use them actually do so” [27]. Our observations also revealed that a number of factors relating to acceptance of improvement initiatives were associated with the social environment. According to the department head of HCO D, it was only when the more senior team members left and two teams were combined that improvement action were able to be implemented in his department. In HCO G, the implementation of a new technogoly was also in opposition to the values of some senior anesthetists. They refused the intrusion of a computer screen during their pre-anesthesia consultations, leaving the onus of subsequent data entry from written notes to others, increasing the likelihood of errors. Moreover, opposition to the new technology was exacerbated by high stress levels due to the departure of the chief anesthetist and ensuing high staff turnover (physicians and nurses). The poor QAF scores in this HCO did not in fact mean, as was initially thought, that clinical managers had not made attempts to undertake improvement initiatives to meet QAF criteria. In different social environments (HCOs A and E), new QAF software for the electronic information system was adapted to user needs before being taken onboard. The recently nominated department head in HCO E did not immediately introduce a compulsory all-electronic anesthesia file but focused first on a single priority step (signatures for discharge from the recovery room) in order not to antagonize the senior anesthetists. Full digitalization was only introduced later.

**Fig. 1:**
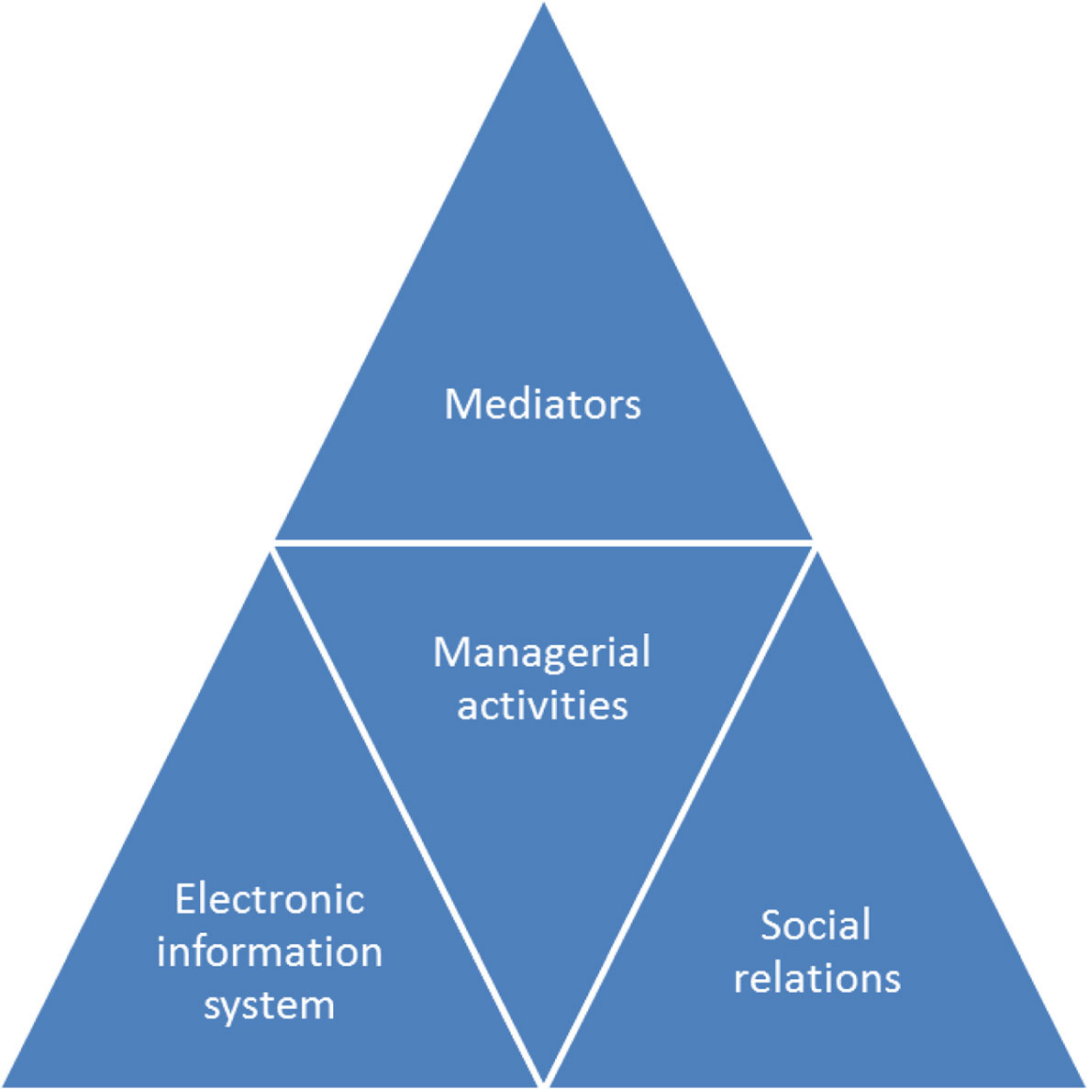
Local managerial activities

Overall, our study revealed that factors related to changes in the electronic information systems and social environment played a role during QAF implementation that were at least as crucial as any action undertaken by the clinical managers. The interaction between these 3 factors – clinical manager involvement, material and social factors – were shown to influence QAF impact on the ground (Fig. 1).

## Discussion

Our study highlights three related findings. First, it has confirmed that a significant proportion of potential QI users, whether anesthetists or other health professionals, are often unaware of quality data [27, 28]. The engagement of team members in improvement actions was provided by the activity of clinical managers (senior anesthetists or heads of department who have professional legitimacy among anesthetists) and was often unnoticed by staff. Studies in the field of implementation sciences often highlight the importance of local leadership involvement in the implementation of quality improvements [29, 30]. Our pragmatic perspective allowed us to specify the nature of this local leadership involvement. The clinical managers have a boundary spanning role, consisting in sharing knowledge across intra-organizational and extra-organizational boundaries [31–34]. They take on the responsibility of a challenging reframing process [35] by translating public policies in terms of local practice. They act as mediators aware of the potentialities of QAF and push the QAF cursor into the realm of clinical practice. Third, our study has emphasized the benefit of considering local managerial activity in terms of the emerging socio-material approach in management science [36, 37, 39]. There was strong overlap between three socio-material constraints, namely, local mediator involvement, coherence of the information system for data collection, and the quality of the interpersonal ties among staff members. Staff climate, the role of information systems, and the potential added value of an electronic information system are each established key factors in quality improvement and safety issues [38, 39]. We have established that local managerial efficacy and QAF implementation requires positive interactions between all three of these factors (see high QAF scores recorded for HCOs A and E). Initiatives undertaken by clinical managers have little chance of success if they come up against unfavorable social environmental factors (see HCO G) [40].

### Implications for policy and practice

Our findings have at least three implications in terms of public management. First, public policies tend to focus on the metrological reliability of QIs as an attribute of the quality mesure [41], on QI relevance to health professionals, and on ranking methods, but tend to overlook local QI implementation. Second, health authorities have chosen to promote local QI implementation by propagating the principles of QI development and the QI scores achieved. However, a public policy is not effective just because it is made known and explained widely. We have clearly shown that widespread knowledge of a QI is not a prerequisite to its implementation. A more effective communication strategy might be for health authorities to target clinical managers who have the legitimacy and clout to put forward improvement initiatives adapted to the local context. Third, local implementation requires the involvement of many professionals. Health authorities give little recognition to the role of clinical managers as mediators both inside and outside the HCO and do not provide them with adequate resources to encourage improvement initiatives. This issue could be addressed by including their role as mediators in their professional development appraisal and by paying for improved quality.

### Study limitations

Our study has the limitations of a qualitative study on a single QI. One limitation was a risk of a social desirability bias because the professionals’ relationship to the QI and its appropriation were discussed during interviews in the anesthesia units [42]. This risk was lessened by systematically cross-referencing the interview data with observations, written documents, official QAF scores as well as data from interviews with staff occupying other posts within the unit. Such cross-referencing showed that high scores were not due to stakeholders being highly committed to quality issues. Staff tended to be honest in their recall of QI use. The scope of our findings might be limited by our choice of a single discipline that, moreover, has long been highly alert to quality issues [43]. According to Hamblin’s typology, the positive conduct of anesthetists is more of the ‘saints” and “honest triers” type than the “reactive gamers” and “rational maniacs” type [44]. Studies in disciplines where the link between QIs and stakeholders is more complex, which relate to more than one discipline, and which involve professionals less well versed in quality issues are needed.

## Conclusions

This study highlights the strong overlap between three key factors (coherence of information systems, fine-tuning of the QI to clinical practice by clinical managers, and the socio-material context) in the implementation of a national QI. In light of our findings, public policies for national QIs should consider not only metrological QI validation but also the context of QI implementation and the crucial mediatory role of clinical managers.
